# Identification of existing pharmaceuticals and herbal medicines as inhibitors of SARS-CoV-2 infection

**DOI:** 10.1073/pnas.2021579118

**Published:** 2021-01-15

**Authors:** Jia-Tsrong Jan, Ting-Jen Rachel Cheng, Yu-Pu Juang, Hsiu-Hua Ma, Ying-Ta Wu, Wen-Bin Yang, Cheng-Wei Cheng, Xiaorui Chen, Ting-Hung Chou, Jiun-Jie Shie, Wei-Chieh Cheng, Rong-Jie Chein, Shi-Shan Mao, Pi-Hui Liang, Che Ma, Shang-Cheng Hung, Chi-Huey Wong

**Affiliations:** ^a^Genomics Research Center, Academia Sinica, Taipei 115, Taiwan;; ^b^School of Pharmacy, National Taiwan University, Taipei 110, Taiwan;; ^c^Institute of Chemistry, Academia Sinica, Taipei 128, Taiwan;; ^d^Department of Chemistry, The Scripps Research Institute, La Jolla, CA 92037

**Keywords:** SARS-CoV-2, drug repurposing, antiviral, cell-based and animal studies

## Abstract

The outbreak of COVID-19 caused by SARS-CoV-2 has resulted in more than 50 million confirmed cases and over 1 million deaths worldwide as of November 2020. Currently, there are no effective antivirals approved by the Food and Drug Administration to contain this pandemic except the antiviral agent remdesivir. In addition, the trimeric spike protein on the viral surface is highly glycosylated and almost 200,000 variants with mutations at more than 1,000 positions in its 1,273 amino acid sequence were reported, posing a major challenge in the development of antibodies and vaccines. It is therefore urgently needed to have alternative and timely treatments for the disease. In this study, we used a cell-based infection assay to screen more than 3,000 agents used in humans and animals, including 2,855 small molecules and 190 traditional herbal medicines, and identified 15 active small molecules in concentrations ranging from 0.1 nM to 50 μM. Two enzymatic assays, along with molecular modeling, were then developed to confirm those targeting the virus 3CL protease and the RNA-dependent RNA polymerase. Several water extracts of herbal medicines were active in the cell-based assay and could be further developed as plant-derived anti–SARS-CoV-2 agents. Some of the active compounds identified in the screen were further tested in vivo, and it was found that mefloquine, nelfinavir, and extracts of *Ganoderma lucidum* (RF3), *Perilla frutescens*, and *Mentha haplocalyx* were effective in a challenge study using hamsters as disease model.

## Significance

COVID-19 is a global pandemic currently lacking an effective cure. We used a cell-based infection assay to screen more than 3,000 agents used in humans and animals and identified 15 with antiinfective activity, ranging from 0.1 nM to 50 μM. We then used in vitro enzymatic assays combined with computer modeling to confirm the activity of those against the viral protease and RNA polymerase. In addition, several herbal medicines were found active in the cell-based infection assay. To further evaluate the efficacy of these promising compounds in animal models, we developed a challenge assay with hamsters and found that mefloquine, nelfinavir, and extracts of *Ganoderma lucidum* (RF3), *Perilla frutescens*, and *Mentha haplocalyx* were effective against SARS-CoV-2 infection.

The severe acute respiratory syndrome coronavirus 2 (SARS-CoV-2) is an enveloped, positive-sense, single-stranded RNA coronavirus of the betacoronaviridae family (1), and the pathogen is responsible for the global pandemic that causes the coronavirus-induced disease in 2019 (COVID-19). Compared to the SARS-CoV and Middle East respiratory syndrome coronavirus (MERS-CoV) outbreaks in 2002 and 2012, respectively, SARS-CoV-2 shows a lower fatality rate, but a much higher transmission rate, causing a greater threat to the public health and extraordinary social and economic burdens (2).

Infection of SARS-CoV-2 starts with the interaction of trimeric viral spike (S) protein with human angiotensin-converting enzyme 2 (ACE2) receptor on airway epithelial cells, followed by viral entry and priming of human transmembrane protease serine 2 (TMPRSS2) that cleaves the S protein and initiates viral fusion (*SI Appendix*, Fig. S1) (3). After entry, the viral genomic RNA is translated to polyprotein 1a (PP1a) and polyprotein 1ab (PP1ab), which are subsequently cleaved by a papain-like (PL) protease and a 3C-like (3CL) protease to form 16 nonstructural proteins (Nsp1-16) as a replication-transcription complex. Four structural proteins (spike, envelope, membrane, and nucleocapsid) are encoded at the 3′ end and play important roles in virus maturation and infection. Replication of viral RNA from the N to C termini of PP1ab is accomplished by replication-transcription complex proteins, such as RNA-dependent RNA polymerase (Rdrp, Nsp12). The viral proteins further undergo posttranslational modifications (such as glycosylation) at the endoplasmic reticulum (ER)-Golgi intermediate compartment, after which they are transported to the cell membrane for exocytosis (4).

To date, the clinical management of COVID-19 is mostly based on supportive care, although several agents targeting viral replication and inflammation have been reported (*SI Appendix*, Fig. S1). Remdesivir, an Rdrp prodrug inhibitor, is the only antiviral agent approved by the US Food and Drug Administration for the treatment of COVID-19 (5). Favipiravir, an inhibitor of influenza Rdrp, was used for the treatment of COVID-19 in Russia, China, and India, but patients receiving the drug must be closely monitored to prevent adverse events; recently, the result of a phase 3 trial in Japan showed some positive effect. Hydroxychloroquine, especially in combination with a zinc supplement, has been reported to exhibit antiviral activity against RNA viruses, but the clinical use of hydroxychloroquine alone for the treatment of COVID-19 was halted due to a lack of significant benefit (6).

It is well known that RNA viruses have higher mutation rates than DNA viruses. Recently, a protein interaction map revealed 332 human proteins interacting with 27 SARS-CoV-2 proteins (7), and a phosphoproteomic approach was further employed to expand the study of viral–host interaction (8). However, the proteomic analysis reported recently was only focused on the S protein and the detailed functions of glycosylation remained unclear (9). Nevertheless, the S protein is a promising target for development of neutralizing antibodies and vaccines due to its expression on the viral surface and its involvement in host cell entry (10). The S protein is highly glycosylated and broadly mutated, with ∼90% of the sequence being changed, indicating the challenge in the development of effective vaccines or antibodies with broadly protective activities and the need to develop alternative therapies (11). However, development of new therapeutics often takes years; therefore, repurposing or repositioning of existing pharmaceuticals and herbal medicines for the treatment of COVID-19 has been considered as an attractive approach.

In this study, we screened a library of 2,855 drugs approved for the treatment of human and animal diseases, as well as 190 supplements and traditional Chinese herbal medicines to identify the inhibitors of SARS-CoV-2 infection to Vero E6 cells. The effective compounds identified from the screening were further studied to establish the dose–response relationship. The compounds that target the proteolytic process, RNA replication, and glycosylation were collected and further evaluated by a target enzyme assay and computer simulation to generate a better understanding of their mode of action. Several active compounds and herbal extracts identified from the cell-based and enzyme-based assays were further evaluated in vivo for their antiinfective effects in hamsters infected with the virus. This investigation identified several promising candidates with potential for further development.

## Results and Discussions

### Cell-Based Screening.

**Fig. 1.**
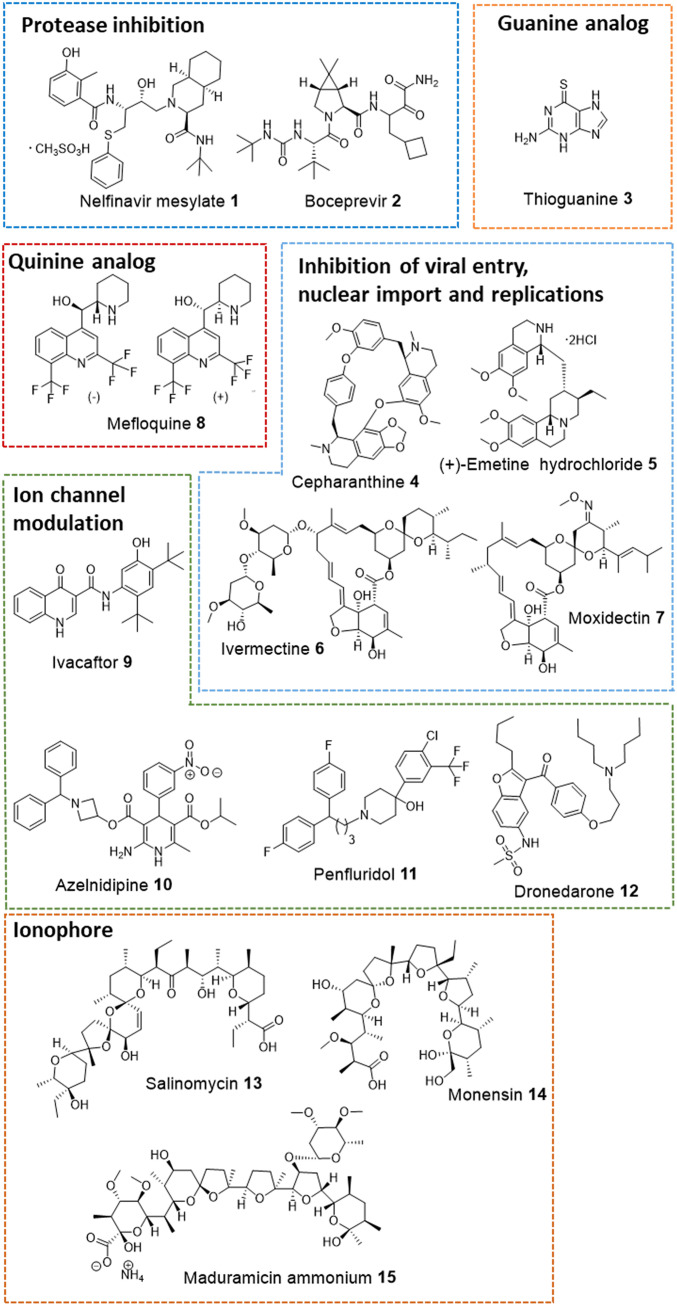
Representative drugs showed antiinfective effects at 10 μM. These drugs are categorized according to their potential mode of action against SARS-CoV-2.

**Fig. 2.**
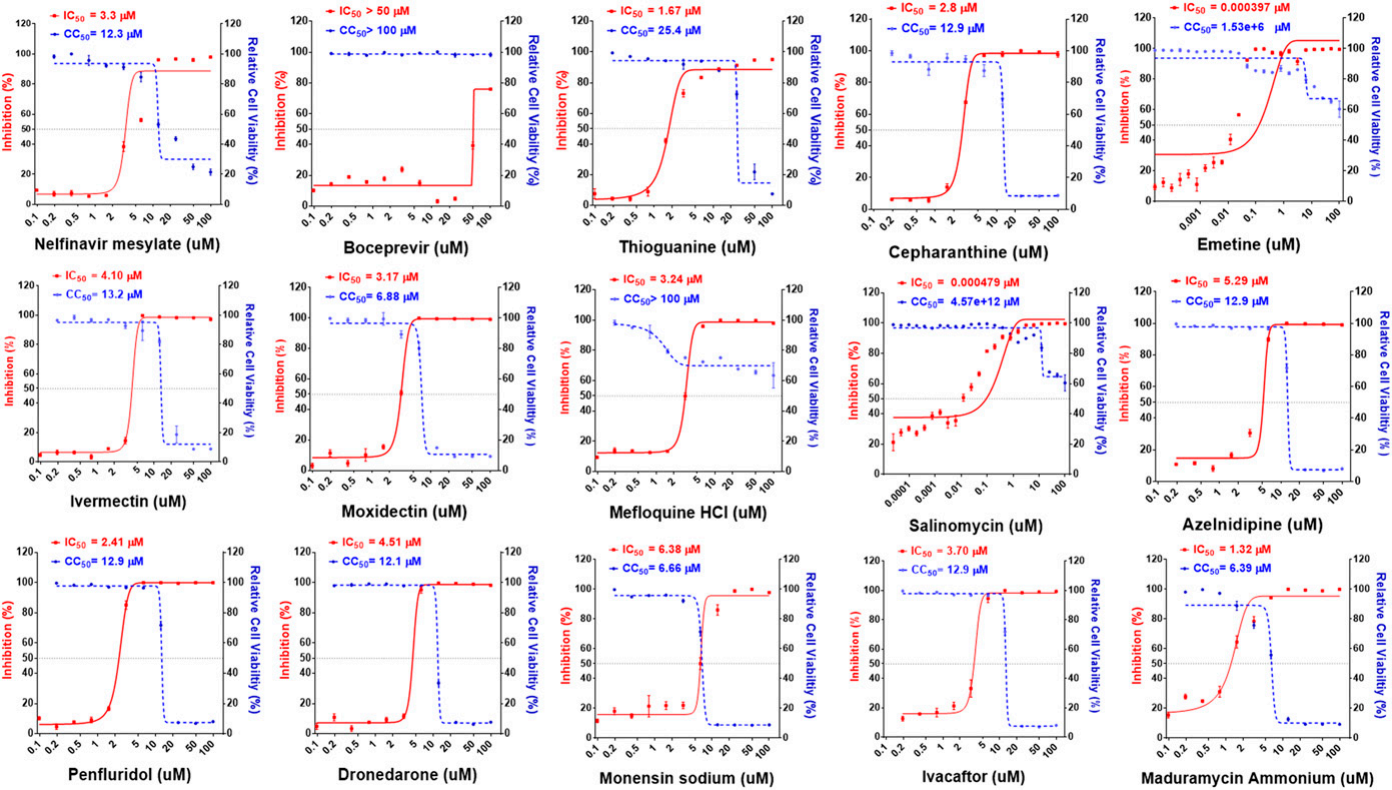
Dose–response relationships of 15 selected antiviral compounds. Vero E6 cells were pretreated with compounds at indicated doses followed by SARS-CoV-2 infection for 48 h. The percentage of viral titer determined by antinucleocapsid antibody after drug treatment (red) and cell viability (blue) were measured and expressed as mean ± SD of at least three independent experiments.

Initially, the antiviral activity of compounds was assessed as previously described (12) by visualization of the extent of cytopathogenic effect (CPE) on Vero E6 cells when infected with a strain of SARS-CoV-2 from Taiwan Centers for Disease Control (hCoV-19/Taiwan/4/2020, isolated from the throat swab of a confirmed 39-y-old male patient from Taiwan) at concentrations of 10 μM, 3.3 μM, and 1 μM, respectively (or in the range from 1 nM to 100 nM for potent compounds). Vero E6 cells are African green monkey kidney epithelial cells that are stable cell lines expressing a high level of the ACE2 receptor and have been used for SARS-CoV research extensively since 2003 (12). In recent study, they have been used in evaluating SARS-CoV-2 infection and replication by measuring viral-induced CPE (3, 13). Of the 2,855 compounds tested, 15 were found to exhibit antiinfective effects in Vero E6 cells and their structures are shown in Fig. 1. The activities of these compounds were evaluated on days 3 and 5, and the minimal concentrations that showed antiinfective effect were recorded (Table 1). The concentrations of an agent required to inhibit 50% (IC_50_) of virus replication and its 50% cytotoxicity (CC_50_) are shown in Fig. 2 and Table 1.

**Table 1.** In vitro anti–SARS-CoV-2 assay

Name	Drug class	Drug indication	CPE (μM)*		IC_50_ (μM)^†^	CC_50_ (μM)^‡^
			D3	D5		
Nelfinavir **1**	Antiviral agent	Anti-HIV infection	5.0	10.0	3.3	12.3
Boceprevir **2**	Antiviral agent	Anti-HCV infection	10.0	10.0	50.1	>10
Thioguanine **3**	Antineoplastic agent	Anticancer	1.25	2.5	1.7	25.4
Cepharanthine **4**	Antineoplastic agent	Anticancer	3.75	7.5	2.8	12.9
Emetine **5**	Antiprotozoal	Antiamoebic	5.0	10.0	4.0e-4	>10
Ivermectin **6**	Anthelmintic	Antiparasitic	2.5	—^§^	4.1	13.2
Moxidectin **7**	Anthelmintic	Antionchoceriasis infection	5.0	10.0	3.1	6.9
Mefloquine **8**	Antimalaria	Prevention and treatment of malaria	5.0	10.0	3.2	>10
Ivacaftor **9**	CFTR potentiators	Cystic fibrosis	5.0	N.I.	3.7	12.9
Azelnidipine **10**	Calcium channel blocker	Antihypertension	5.0	N.I.	5.3	12.9
Penfluridol **11**	First generation antipsychotic	Schizophrenia	5.0	10.0	2.4	12.9
Dronedarone **12**	Ion channel blocker	Cardiac arrhythmia	7.5	N.I.	4.5	12.1
Salinomycin **13**	Polyether antibiotic	Prevent coccidiosis of animals	0.039	0.156	4.8e-4	13.1
Monensin **14**	Polyether antibiotic	Prevent coccidiosis of animals	0.117	2.5	6.4	6.6
Maduramicin **15**	Polyether antibiotic	Prevent coccidiosis of animals	0.039	0.117	1.3	3.4

CTFR, cystic fibrosis transmembrane conductance regulator; N.I., no inhibition.

* The minimal dose that showed antiinfective effect after virus challenge after incubation for 72 h (D3) and 120 h (D5).

† 100 PFU of SARS-CoV-2 (multiplicity of infection 0.025) in Vero E6 cells.

^‡^ Vero E6 cells.

^§^ Cytotoxicity was observed.

### Protease Inhibitors.

To understand the mode of action, some active compounds were subjected to target-based assay, and the two major proteases of SARS-CoV-2, PL protease and 3CL main protease, were chosen as targets. When tested at 100 μM, dronedarone (**12**) showed a significant inhibition against PL protease, while the other two known protease inhibitors [nelfinavir mesylate (**1**) and boceprevir (**2**)] showed inhibition against the 3CL protease (*SI Appendix*, Table S1). The inhibition of substrate cleavage by the enzyme was further confirmed by the HPLC analysis of cleaved substrate (*SI Appendix*, Fig. S5).

Nelfinavir mesylate (**1**) is an inhibitor of HIV protease with proven efficacy as an inhibitor of SARS-CoV replication (14). In our assay, nelfinavir mesylate (**1**) showed inhibition of SARS-CoV-2 replication with IC_50_ of 3.3 μM (Table 1). Although the clinical result of lopinavir and ritonavir combination therapy showed no significant benefits and lowered the anticipation of drug repurposing with HIV protease inhibitors (15, 16), nelfinavir was found in this study to be active in the cell-based and target-based assays and is one of the few compounds achieving higher plasma concentration than the reported ones in the normal dosing interval (17). The library used in the screen also contains several other clinically approved HIV protease inhibitors, including indinavir sulfate, saquinavir mesylate, atazanavir, ritonavir, darunavir, amprenavir, and lopinavir, and the hepatitis C virus (HCV) protease inhibitors daclatasvir, danoprevir and telaprevir, but none of these showed inhibition against SARS-CoV-2 in the concentrations used in the screen, except boceprevir (**2**), which displayed a relatively weak activity (IC_50_ of 50.1 μM) (Fig. 2 and Table 1).

**Fig. 3.**
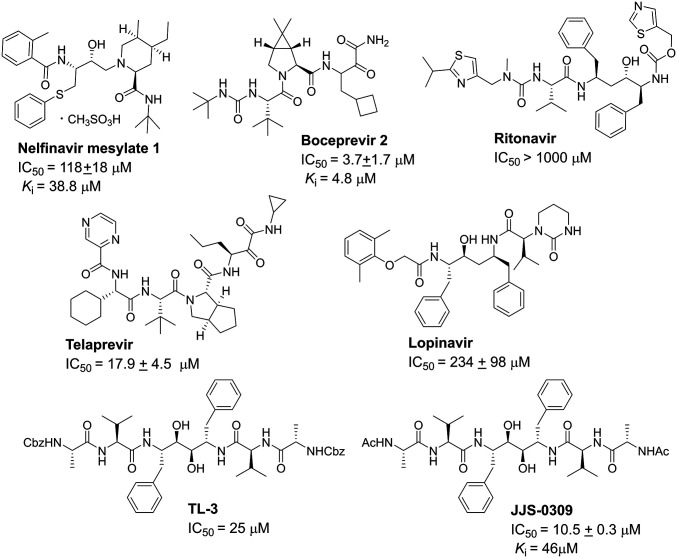
Structures of protease inhibitors and their IC_50_ and *K*_*i*_ values for 3CL protease inhibition. The values were determined from three independent experiments using FRET-based enzymatic assays.

The inhibition of nelfinavir mesylate and boceprevir, along with other HIV protease inhibitors, against the SARS-CoV-2 3CL protease (nsp 5) was measured in vitro subsequently to determine the *K*_*i*_ values, and the result showed that boceprevir (*K*_*i*_ = 4.8 μM) (Fig. 3 and *SI Appendix*, Fig. S5) is more potent than nelfinavir mesylate (*K*_*i*_ = 38.8 μM). We also tested JJS-0309, a TL-3 derivative active against the protease of feline immunodeficiency virus, HIV, and SARS-CoV (18), but it did not show any inhibition against SARS-CoV-2 in the cell-based assay, indicating the differences between the two SARS proteases (Fig. 3).

**Fig. 4.**
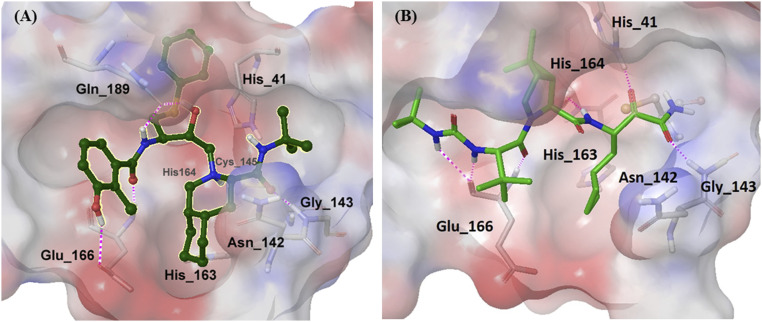
Computer simulation of (*A*) nelfinavir and (*B*) boceprevir binding to SARS-CoV-2 3CL protease (PDB ID code 6LU7). Pink dashed lines indicated interaction between compounds and protein.

The inconsistency between the *K*_*i*_ value and the antiviral efficacy in the cell-based assay for nelfinavir mesylate and boceprevir prompted us to evaluate their target binding with computer modeling. The result of molecular docking showed that the interactions between nelfinavir mesylate (*K*_*i*_ of 38.8 μM) and SARS-CoV-2 3CL protease were mainly through residues Gly143, Glu166, and Gln189 (Fig. 4*A*), and as for boceprevir (*K*_*i*_ of 4.8 μM), more interactions were observed with His41, Gly143, His164, and Glu166 residues (Fig. 4*B*), consistent with the observed higher inhibition activity in the enzyme assay. However, nelfinavir mesylate exhibited 15-fold higher anti–SARS-CoV-2 activity than boceprevir in the cell-based assay, probably due to its inhibition of multiple targets or the differences in cell permeability or in cell fusion caused by the S protein (19).

### Guanine Analog.

Thioguanine (**3**), an antimetabolite used for the treatment of cancers and autoimmune diseases, showed an IC_50_ of 1.7 μM. Since it is an analog of guanine, it is expected to act as a prodrug and after conversion into thioguanosine triphosphate (TGTP), it could act as an inhibitor of Rdrp or GTP-binding protein associated with cellular DNA synthesis and replication (20). The molecular docking also indicated that TGTP fits well with Rdrp (*SI Appendix*, Fig. S3). TGTP was therefore synthesized (*SI Appendix*, Fig. S6) and tested as an inhibitor of Rdrp in vitro; unfortunately, there was no inhibition against Rdrp. In previous reports, thioguanine was found to act as a slow-binding, reversible, and competitive PLpro inhibitor of SARS-CoV (21) and SARS-CoV-2 (22), indicating further study was necessary to understand the mechanism of thioguanine and thioguanine analogs against SARS-CoV-2. In addition, several known viral polymerase inhibitors—including acyclovir, famciclovir, penciclovir, ribavirin, cidofovir, and entecavir—and reverse-transcriptase inhibitors—including zalcitabine, nevirapine, efavirenz, abacavir sulfate, tenofovir disoproxil fumarate, adefovir dipivoxyl, delavirdine, and telbivudine—were also screened in the cell-based assay but no active compound was identified.

### Spike Protein Mutation and Glycosidase Inhibitors.

**Fig. 5.**
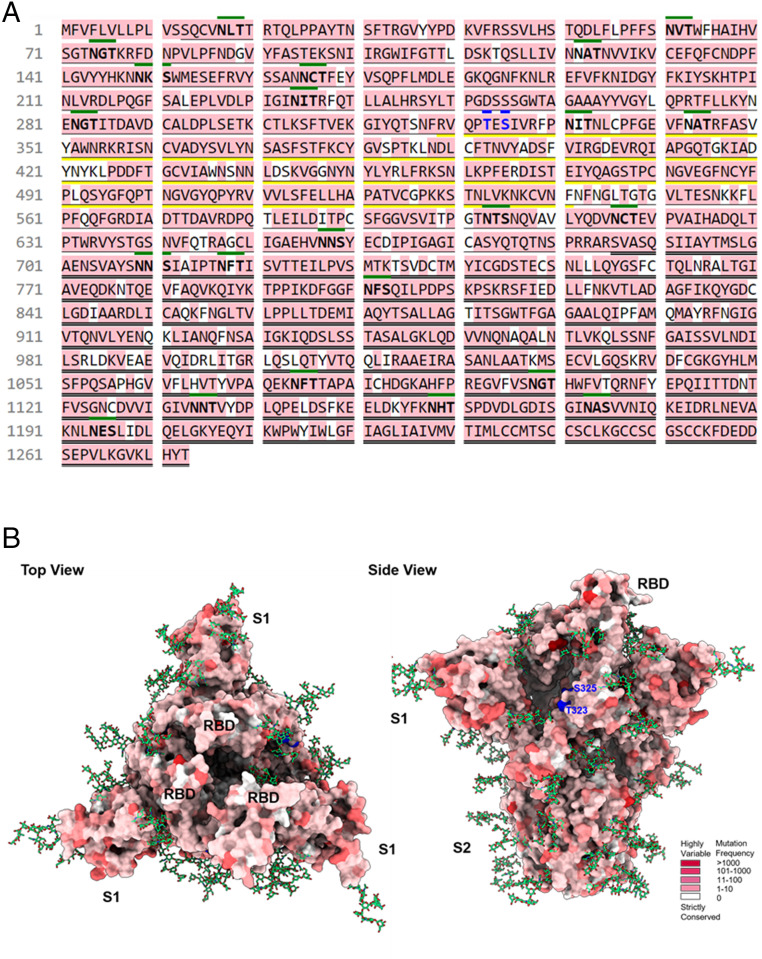
SARS-CoV-2 spike protein sequence mutation analysis. (*A*) Analysis of spike protein mutation from the 196,276 sequences revealed 1,141 sites of mutation in the 1,273 amino acids of spike protein. The spike protein sequence of hCoV-19/Taiwan/4/2020 used in this study was identical to the original virus strain (UniProt Entry: P0DTC2). Single bottom line: S1 region (residues 13 to 685); double bottom line: S2 region (residues 686 to 1273); yellow bottom line: receptor binding domain (residues 319 to 541); green top line: *N*-glycosylation motifs; blue top line: *O*-glycosylation sites; pink: mutation residues; sequence representative: P0DTC2 (UniProt Entry). (*B*) Top and side view of the S protein indicating residue variants (from strictly conserved to highly variable: white to red), *N*-glycans (green stick), *O*-glycosylation sites (blue) in three-dimensional structure (PDB ID code 7CN9).

Analysis of the 196,276 sequences of S protein variants obtained from the GISAID (Global Initiative on Sharing Avian Influenza Data) database (23) on November 15, 2020 revealed that the S protein has already mutated on 1,141 sites of the 1,273 amino acid sequences (i.e., ∼90% of the S protein has been mutated) (Fig. 5). In addition, the trimeric S protein is highly glycosylated with 22 *N*-glycosites and 2 *O*-glycosites per monomer (9, 24); the 2 *O*-glycosites and 2 *N*-glycosites are located in the receptor binding domain (RBD) region, while some other *N*-glycosites are fully conserved (e.g., N122, N343). However, glycosylation is cell-specific and the role of glycosylation in S protein during viral infection and interaction with other host proteins in airway epithelial cells is not well understood and could be a subject for further investigation. Nevertheless, despite the broad scope of mutation in the S protein, it appears that most variants still interact with ACE2, suggesting that the RBD domain may have little change in conformation and ACE2 could serve as a decoy to block the entry of the virus.

In any event, disruption of the glycosylation process of S protein by α-glucosidases I and II could lead to the formation of incorrect glycoform, causing protein misfolding, ER-associated degradation, interference of protein–protein interaction, and inhibition of virus maturation (25). α-Glucosidase was a promising anti–SARS-CoV-2 target since the production of endo-α-1,2-mannosidase (MANEA) was not detectable in human pneumocytes (26), and a deep mutational scanning study revealed that the N343 glycosylation was necessary for the RBD expression (27). In addition, a computer simulation of S protein also suggested the importance of *N*-glycans at N165 and N234 in modulating the receptor binding of S protein (28). However, among the five α-glucosidase inhibitors (miglitol, voglibose, acarbose, *N*-methyl-1-deoxynojirimycin, daucosterol) in our library and several iminocyclitols from previous studies (*SI Appendix*, Fig. S4) evaluated, none was able to efficiently block the viral infection in the cell-based assay. This preliminary result seems to suggest that the role of glycosylation in the cell-based infection process is not significant, although the assay may not represent the complete picture of the virus infection cycle, or the lack of activity may be due to the compensating effect of MANEA in Vero E6 cells after inhibition of glucosidase (29), or due to the different natures of cells and virus used in the assay (30).

### Inhibitors of SARS-CoV-2 Entry, Nuclear Import, and Replication.

Cepharanthine (**4**, IC_50_ of 2.8 μM) is an alkaloid isolated from *Stephania*, exhibiting multiple biological efficacies and rare adverse events. It was used for leukopenia, snake bites, and alopecia, and was reported to have antiviral activities with multiple mechanisms in laboratory research (31). Recently, it was found to be able to block virus entry and show a synergistic antiviral activity with nelfinavir (32). (+)-Emetine hydrochloride (**5**, IC_50_ of 0.00040 μM) is an antiprotozoan agent approved for amoebiasis. Emetine was found to be a broad-spectrum inhibitor of other coronaviruses (33), and it was also identified in this screen as an effective inhibitor of SARS-CoV-2 replication. The cardiotoxicity of emetine could be a concern, and whether it could be used at low dose or in combination with other antiviral agents, such as remdesivir, to achieve better clinical benefits remained to be investigated (34).

Ivermectin (**6**, IC_50_ of 4.1 μM) and moxidectin (**7**, IC_50_ of 3.1 μM) share the same scaffold and are potent avermectin-type compounds that have been used as antiparasitic agents. Their mode of action is through the enhancement of glutamate-gated chloride channel, leading to chloride influx and paralyzing the parasite. One of the antiviral mechanisms was related to the inhibition of proteins associated with viral nuclear import (35). However, a study predicted with a population pharmacokinetic model indicated that the concentration of ivermectin in the lung might not achieve the desired level with even 10 times of approved dosing regimen (36). Both ivermectin and moxidectin exhibited high cytotoxicity in the cell-based assay.

Mefloquine (**8**) belongs to the class of quinine-type antimalarial agents, and is effective against SARS-CoV-2 infection with IC_50_ of 3.2 μM. During the pandemic, the antimalarial agents chloroquine and hydroxychloroquine (also used for the treatment of autoimmune diseases) were reported to exhibit promising anti–SARS-CoV-2 effect in an early in vitro study (37), leading to large clinical trials to evaluate the benefit of chloroquine and hydroxychloroquine in patients. Although quinine-type compounds seemed to have some positive activities against SARS-CoV-2 infection, the cardiac side effects of chloroquine and hydroxychloroquine in clinical studies were reported (6). Thus quinine-type compounds with lower cardiac toxicity and cytotoxicity, such as mefloquine, could be explored as an alternative candidate.

### Ion Channel Modulator and Ionophores.

Four compounds (ivacaftor **9**, IC_50_ = 3.7 μM; azelnidipine **10**, IC_50_ = 5.3 μM; penfluridol **11**, IC_50_ = 2.4 μM; dronedarone **12**, IC_50_ = 4.5 μM) identified in our library with anti–SARS-CoV-2 activities were categorized into ion channel modulators. Electrolyte homeostasis is an important factor for viruses to replicate and survive, and many of them expressed viroporins to control host ion balance (38). Since the ion channel displays various roles in the viral life cycle, such as virus entry and replication, channel modulators are a new type of broad-spectrum antiviral agents (39). However, their potential toxicity remained to be concerned.

Three compounds used as animal-ionophoric antibiotics to prevent coccidiosis of chicken were identified in this study to be effective against SARS-CoV-2. Monensin (**14**) exhibited an IC_50_ of 6.4 μM and maduramicin ammonium (**15**) has an IC_50_ of 1.3 μM, but both of them showed no selectivity between the antiviral effect and cytotoxicity. However, salinomycin (**13**) exhibited an excellent antiviral activity (IC_50_ of 0.00048 μM) and selectivity (CC_50_ of 13.1 μM, selectivity index > 100). The mode of action for salinomycin is still unclear, but it was proposed to disrupt the endosomal acidification and to block the entry of viruses into cell and enhance host-directed antiviral responses (40). Autophagy was recently identified to play a role in host antiviral immunity, and was effective in the inhibition of coronaviruses (41). Ionophoric compounds were reported to trigger autophagy through inducing an electrolyte imbalance in potassium and sodium (42), and inhibition of E3-ligase S-phase kinase-associated protein 2 (SKP2) (41). Since hypokalemia and hyponatremia were common in severe SARS-CoV-2 patients (43), the therapeutic window of salinomycin and its combination with an electrolyte supplement, such as Zn-gluconate, could be further evaluated.

### Traditional Chinese Herbal Medicines.

**Fig. 6.**
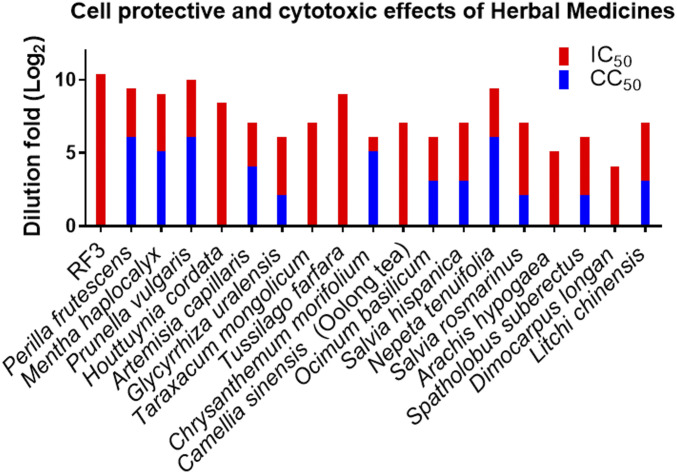
Evaluations of antiviral effect of Chinese herbal medicines in serial dilutions were presented as log_2_(dilution fold). Anti–SARS-CoV-2 infection effects of selected Chinese herbal medicines as water extracts (1.0 g/20 mL H_2_O) and RF3 dissolved in H_2_O (0.25 mg/mL) are presented. The tested results of all Chinese herbal medicines are summarized in *SI Appendix*, Fig. S7.

Some well-known traditional Chinese herbal medicines were also tested in the cell-based assay (Fig. 6 and *SI Appendix*, Table S2). The medical herbs (1.0 g) were extracted by water (20 mL) at room temperature and diluted with the assay buffer. We found that the aqueous extracts of herbs from Lamiaceae (*Perilla frutescens*), Mentheae (*Mentha haplocalyx*), Asteraceae (*Taraxacum mongolicum*, *Tussilago farfara*, *Chrysanthemum morifolium*), Theaceae (*Camellia sinensis*), Lamiaceae (*Prunella vulgaris*, *Ocimum basilicum*, *Salvia hispanica*, *Nepeta tenuifolia*, *Salvia rosmarinus*), Fabaceae (*Arachis hypogaea*, *Spatholobus suberectus*), and Sapindaceae (*Dimocarpus longan*, *Litchi chinensis*) families were able to reduce the CPE of SARS-CoV-2 in Vero E6 cells when the extracts were diluted to 16- to 960-fold (Fig. 6). These herbs contain flavonoids (myricetin from litchi chinensis seed) (44), flavan-3-ol (catechin and epigallocatechin gallate from tea and Spatholobus root) (45, 46), caffeic acid derivatives [caftaric acid and chlorogenic acid (47) from purple coneflower or honeysuckle flower bud or chia seed (48)] (*SI Appendix*, Fig. S2), and whether they are related to the antiviral activity remains to be investigated. It was reported that monoterpenes (1, 8-cineole and camphor from basil leaves) (49), diterpenes [carnosic acid (50) and patchouli alcohol (51) from rosemary, and patchouli], and triterpenes (ursolic acid from prunella spike and basil) were able to block virus entry and replication. However, the exact mechanisms of these Chinese herbal medicines to inhibit the infection of SARS-CoV-2 are still unknown, and synergistic effects of multiple active components may exist (52).

On the other hand, several fractions of l-fucose–containing polysaccharides previously isolated and characterized from *Ganoderma lucidum* (Reishi) were tested in the cell-based anti–SARS-CoV-2 assay, and the Reishi l-fucose–containing polysaccharides fraction 3 (RF3) was found to exhibit outstanding antiviral efficacy (2 μg/mL), and it was still active at 1,280-fold dilution, with no cytotoxicity (Fig. 6) (53). Although the preliminary results from cell-based experiments cannot be directly extrapolated to clinical outcomes, the potential of RF3 as anti–SARS-CoV-2 agent is worth of further evaluation.

### In Vivo Anti–SARS-CoV-2 Assay.

**Fig. 7.**
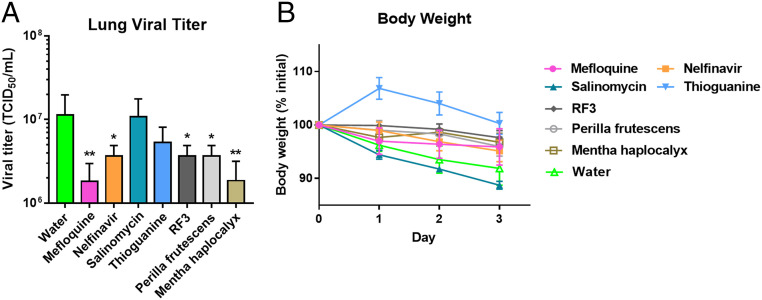
In vivo anti–SARS-CoV-2 assay conducted in female golden Syrian hamsters. (*A*) Virus elimination effect of drugs and extracts. Hamsters were infected with SAR-CoV-2 by intranasal instillation at day 0, and treated with drugs and extracts orally twice a day (30 mg/kg/d for drugs and 200 mg/kg/d for extracts) continuously for 3 d. After 3 d, the lungs were collected to measure the virus load (*n* = 5), *P < 0.05; **P < 0.005. (*B*) Body-weight change after 3-d treatment, *n* = 5 for test group and *n* = 6 for the control group.

Four of 15 active drugs identified from the cell-based assay—mefloquine, nelfinavir, salinomycin, and thioguanine—were selected to evaluate their antiinfective efficacy in female golden Syrian hamsters. Among the active herbal medicines, three extracts—RF3, *P. frutescens*, and *M. haplocalyx*—were also selected for further evaluation since RF3 exhibited a significant antiinfective/cytotoxic selectivity, and *P. frutescens* and *M. haplocalyx* were major active gradients in RespireAid TM (NRICM101). Salinomycin was shown to cause higher weight loss than the control group (water administrated), while all other drugs and extracts showed insignificant weight loss. Therefore, the issue of acute toxicity was not a concern in the chosen drugs and extracts, except for salinomycin (Fig. 7*B*). In the animal study, hamsters were infected with SARS-CoV-2 intranasally at day 0, and after 3-d treatment of orally administered drugs (at a dose of 30 mg/kg/d) and extracts (200 mg/kg/d) the hamsters were sacrificed and the lungs were collected to measure the viral load. Surprisingly, the two compounds, thioguanine and salinomycin, with better cell-based assay activities showed no significant antiinfective effect in the animal study. However, mefloquine and extract of *M. haplocalyx* significantly reduced viral load than that of control (*P* = 0.005) (Fig. 7*A*), and nelfinavir, extracts of RF3, and *P. frutescens* also showed good antiviral effects (*P* = 0.03 v.s. control) (Fig. 7*A*). Overall, in the in vivo assay, mefloquine and nelfinavir were identified as potential drug-repurposing agents and extracts of *M. haplocalyx*, *P. frutescens*, and RF3 showed potential as anti–SARS-CoV-2 herbal candidates.

The inconsistency of the antiviral efficacy in cell and animal assays may originate from the high hydrophilicity and low oral bioavailability of compounds, or from the different infection mechanisms in the assay models, or the different immune systems of animals. Golden Syrian hamsters were shown to be better than mice as animal models for SARS-CoV-2 infection since the ACE2 transgenic mice were not readily available (54), and the hamster model was shown to have similar histopathological properties to humans in upper and lower respiratory tract infections and coherent inflammatory cytokines profiles (55). However, viral clearance was observed in hamsters after day 6 postinfection, indicating that hamsters may only mimic the mild human COVID-19 case (56). A mouse-adapted SARS-CoV-2 strain was reported and demonstrated to represent more severe infection, but the preparation was time-consuming and the mutation in the receptor-binding domain might alter the function of spike protein (57). *Rhesus macaque* seems to be a good model for the development of anti–SARS-CoV-2 agents, as it contains similar anatomy, physiology, and immune systems to that of humans. However, this model is limited by the availability and cost, and it cannot represent the severe case in humans (58). Therefore, developing an appropriate and accessible animal model to mimic the complete human infection process is an urgent need for accelerating the development of anti–SARS-CoV-2 agents.

## Conclusion

In summary, 15 chemical entities from a library of 2,855 compounds approved for human or animal use have been identified in this Vero E6 cell-based study to have the anti–SARS-CoV-2 activity. These compounds were categorized into five groups as viral protease inhibitors (nelfinavir, boceprevir), guanine analog (thioguanine), inhibitors of SARS-CoV-2 function (cepharanthine, emetine, ivermectin, moxidectin, mefloquine), ion channel modulators (ivacaftor, azelnidipine, penfluridol, dronedarone), and ionophoric antibiotics (salinomycin, monensin, maduramicin). Since the safety and pharmacological characteristics of these drugs were extensively studied, the preclinical and clinical assessments of the active compounds identified in this study are expected to be rapid, and can efficiently reduce the time and cost for further development. In addition, several extracts of Chinese herbal medicines and supplements showed promising anti–SARS-CoV-2 effects in Vero E6 cell-based assays, and of particular significance are the species of Asteraceae, Theaceae, Mentheae, and Lamiaceae family, as well as the RF3 fraction. A recent study showed that heparan sulfate acted as a coreceptor for the S protein (59). However, several heparan sulfate-related structures were shown to be inactive in our cell-based assay, although RF3 exhibited a significant inhibition activity, and its mode of action remains to be investigated. These herbal medicines with anti–SARS-CoV-2 activities could also be interesting sources for the discovery of new chemical entities as inhibitors of the virus. However, due to the lack of precise animal model for further evaluation of promising candidates identified from the cell-based assays, the active agents reported in this study may have to be further assessed when a better animal model is available.

## Materials and Methods

Detailed information on the compound sources, primary screening, IC_50_ of anti–SARS-CoV-2 effect, cytotoxicity in Vero E6 cells, inhibitory activity against SARS-CoV-2 protease and Rdrp, computer modeling of inhibitors binding to SARS-CoV-2 3CL protease and Rdrp, analysis of S protein variants, and animal study are described in *SI Appendix*, *Materials and Methods*.

All experiments involving live SARS-CoV-2 were performed in an animal BSL-3 facility at the Genomics Research Center, Academia Sinica. The study protocol was approved by the Institutional Animal Care and Use Committee.

## Supplementary Material

Supplementary File

## Data Availability

All study data are included in the article and *SI Appendix*.
