# Supplementary material for: Identification of existing pharmaceuticals and herbal medicines as inhibitors of SARS-CoV-2 infection
Source: Proc Natl Acad Sci U S A. 2021 Jan 15;118(5):e2021579118. doi: 10.1073/pnas.2021579118 (PMC7865145; doi:10.1073/pnas.2021579118)
Supplement: Supplementary File [file pnas.2021579118.sapp.pdf]

PNAS
www.pnas.org

Supplementary Information for

Identification of Existing Pharmaceuticals and Herbal Medicines as Inhibitors of SARS-CoV-2 Infection

Jia-Tsrong Jan[a], Ting-Jen Rachel Cheng[a], Yu-Pu Juang[b], Hsiu-Hua Ma[a], Ying-Ta Wu[a], Wen-Bin Yang[a], Cheng-Wei Cheng[a], Xiaorui Chen [a], Ting-Hung Chou[c], Jiun-Jie Shie[c], Wei-Chieh Cheng[a], Rong-Jie Chein[c], Shi-Shan Mao[a], Pi-Hui Liang[a, b, 1], Che Ma[a, 1], Shang-Cheng Hung[a, 1], and Chi-Huey Wong[a, d, 1]

[1]Corresponding authors: phliang@ntu.edu.tw; cma@gate.sinica.edu.tw; schung@gate.sinica.edu.tw; chwong@gate.sinica.edu.tw or wong@scripps.edu;

This PDF file includes:

Supplementary text
Figures S1 to S7
Tables S1 to S2
SI References

Supplementary Information Text

Materials and methods

Compound Library

A total of 2,855 unique molecules or chemical entities, approved and clinically-evaluated drugs were gathered (from various sources, including TargetMol, TimTec, MicroSource Discovery Systems, Inc., and Selleck Chemicals) for the antiviral screening in this study. These compounds were dissolved in DMSO at a concentration of 1 mM and transferred to 96-well microtiter plates for the antiviral activity assay based on the prevention of the SARS-CoV-2-mediated cytopathic effect. 190 herbal medicines and supplements were also collected by the following procedure: dissolving 1.0 gram of the ground herbal medicine in water (20 mL) for 4 hours and the mixture was centrifuged to collect the supernatant. Then, 100 µL of the supernatant were combined with 100 µL of the DMEM medium, and the mixture was used for the assay. Reishi raw materials were obtained from Wyntek and fractionated as previously described (1).

Primary screening for identification of anti-SARS-CoV-2 compounds. Compounds from the library prepared as 1 mM solutions in DMSO were transferred into 96-well plates. All compounds were diluted in DMEM (2% FBS) to final concentrations of 10, 3.3, and 1 µM (or lower conc. for potent compounds) for screening. Herbal extracts (1.0 g/20 mL H_2O) and Reishi extracts (0.25 mg/mL) were 2-fold serial diluted in DMEM (2% FBS) for screening. Vero E6 cells (1×10^4 per well) were cultured in a 96-well plate in DMEM supplemented with 10% FBS. The culture medium was removed after a 1-day incubation, when the cells reached 80–90% confluence. A solution of 100 µL of DMEM, with 2% FBS containing the compound to be tested, was placed in each of three wells. Cells were incubated in a CO_2 incubator at 37°C with a SARS-CoV-2 strain from Taiwan CDC (hCoV-19/Taiwan/4/2020, isolated from the throat swab of a confirmed 39 y/o male patient from Taiwan) at a dose of 100 $TCID_{50}$ per well; the cytopathic morphology of the cells was examined by using an inverted microscope at 72 h and 120 h.

Quantification of SARS-CoV-2 via antibody staining in the presence of inhibitors. Vero E6 cells were seeded into 96-well plates (1×10^4 per well) and incubated for 24 h. Two hours before infection, the medium was replaced with 50 µL of DMEM (2% FBS) containing the compound of interest at concentrations 50% 2-fold greater than those indicated, including a DMSO control. Plates were then transferred into the BSL-3 facility and 100 PFU of SARS-CoV-2 was added in 50 µL of DMEM (2% FBS), bringing the final compound concentration to those indicated. Plates were then incubated for 24 h at 37 ^oC. After infection, the supernatants were removed and cells were fixed with 4% formaldehyde for 24 h prior to removal from the BSL-3 facility. The cells were then immune-stained for the viral NP protein (antisera produced in Ma Che’s lab; 1:3000) with a DAPI

counterstain. Infected cells and total cells (DAPI) were quantified using the Celigo (Nexcelcom) imaging cytometer. Infectivity was measured by the accumulation of viral NP protein in the nucleus of the Vero E6 cells (fluorescence accumulation). Percent infection was quantified and the DMSO control was then set to 100% infection for analysis. The IC_{50} for each experiment was determined using the Prism software (GraphPad 8.0, San Diego, CA, USA).

Cytotoxicity study of compounds with Vero E6 Cells

The cytotoxicities of compounds to the Vero E6 cells were assayed using the CCK-8 cell counting kit (Dojindo Labortories) according to the manufacturer's protocol. Briefly, after incubation with the indicated compounds at various concentrations for 24 h, 10 μL of the CCK-8 reagent were added to each well of the 96-well plate and placed in a CO_2 incubator for 1-4 h to react. The absorbance was measured with a spectrophotometer (SpectraMax M2, Molecular Devices) at 450 nm. Data are expressed as percentage of control cells (as 100%) cultured in the absence of compounds.

Activity assay for SARS-CoV-2 proteases

The protease assays were based on a previously reported method (2). Briefly, the gene encoding the PL protease (nsp3) of SARS-CoV-2 was amplified from its cDNA (MN997409, a gift from National Taiwan University College of Medicine) and expressed as $(His)_6$-tagged protein. The PL protease was expressed from *E. coli* BL21 cells and purified using Ni sepharose (GE healthcare). The gene encoding the 3CL main protease (nsp5) of SARS-CoV-2 was amplified and expressed as a Glutathione S-Transferase (GST) tagged protein, with the FXa cleavage site (IEGR) between GST and the protease gene. The recombinant genes were expressed from *E. coli* BL21 cells, purified with glutathione sepharose 4B (GE Healthcare), and then digested with FXa protease (New England Biolabs) to remove the GST tag. The digestion mixture was then passed through glutathione sepharose 4B and the flow through was concentrated for protease activity studies. All kinetic measurements were performed in 20 mM bis(2-hydroxyethyl)amino]tris(hydroxymethyl) methane (pH 7.0) at 30°C. The enhanced fluorescence due to cleavage of the fluorogenic substrate peptide (FAM-LKGGKIVNK-QXL520-NH_2 for nsp3 and FAM-AVLQSGFRK-QXL520-NH_2 for nsp5) (Anaspec) was monitored at 530 nm with excitation at 480 nm using a fluorescence plate reader (Clariostar, BMG Labtech). A series of concentrations of inhibitors were tested and IC_{50} values /*Ki* values were derived by using GraphPad Prism 7.0 (GraphPad Software, Inc.). Confirmation of protease cleavage of the substrates was conducted by high-performance liquid chromatography on a Zorbax C18 column (with a gradient of 5% to 25% acetonitrile containing 0.1% trifluoroacetic acid) and by mass spectrometry analysis.

Activity assay for SARS-CoV2 RNA-dependent RNA polymerase

The genes encoding nsp7, nsp8 and nsp12 of SARS-CoV-2 were synthesized (a gift from Dr. Wai-Lung Ng, The Chinese University of Hong Kong) and were

expressed as $(His)_6$-tagges proteins from insect cells using multi-cistronic expression. The activity of RNA synthesis was monitored by incubating 5 μM of nsp7-nsp8-nsp12 complex with 5 μM of annealed RNA (5′-fluorescein-UUUUCUACGCGUAGUUUUCUACUGCG-3′) in the buffer containing 20 mM HEPES, pH 7.5, 100 mM NaCl, 5%, glycerol, 10 mM $MgCl_2$, and 5 mM β-mercaptoethanol, 150 μM NTPs for 2 h at 37 °C (3). The reaction mixture was subjected to denaturing at 8 M urea and 20% polyacrylamide gel electrophoresis to resolve products of RNA synthesis followed by analysis of fluorescent image. Alternatively, the synthesized RNA was isolated (RNA Clean & Concentrator Kits, Zymo Research) and subjected to mass spectrometry analysis (Genomics Research Center and Institute of Chemistry, Academia Sinica).

Computer modeling of SARS-CoV-2 3CL protease and RdRp inhibitions.
Molecular docking studies were carried out using GLIDE (Schrödinger, Inc, NY) and the binding free energies were calculated by AutoDock v4.2 (4). Initially, the molecular geometry and atomic coordinates were taken from the crystallographic structure of SARS-CoV 3CL protease (PDB coded 6LU7), which is complexed with a peptidomimetic inhibitor (5) and cryo-EM structure of Rdrp (PDB coded 7BV2) (6). The covalent bond between the Sγ atom of C145 and the inhibitor was removed and the Sγ position was adjusted to maintain the re-docked pose of the original inhibitor without disturbing by the atoms. Since the remdesivir-enzyme complex in the cryo-EM was present in its monophosphate form with a diphosphate product, RTP molecular structure was regenerated in the complex (PDB coded 7BV2), having the two canonical magnesium ions adjusted to locate in an active form with the triphosphate group (7). The new RTP-RdRP complex structure was compared with the RNA polymerases of HIV (PDB code 1rtd), poliovirus (PDB code 1rdr), and ZIKA (PDB code 5tfr) for the positions of the triphosphate group and the two magnesium ions. The compound structures were generated and optimized by LigPrep (Schrödinger, LLC, NY) with OPLS3 (8) and Epik (9). Before docking, the protein structures were refined by Protein Preparation Wizard (Schrödinger, LLC, NY) and the docking grids were generated around a 20 × 20 × 20 box centered at the catalytic sites represented by Cys145 for 3CL protease and Glu760 for Rdrp.

Analysis of the S protein variants.
The 200,619 S-protein sequences of SARS-CoV-2 were downloaded from the GISAID database (version: Nov. 15, 2020). Only protein sequences from human samples with length 1,273 were kept for multiple sequence alignment (MSA, total 196,276 sequences). MSA was run by MAFFT program (version: 7.474) (10) with FFT-large-NS-2 alignment strategy. The mutation information from the MSA result and other sequence information were annotated and visualized in Figure 5A by our in-house Python program. The 3D structure of SARS-CoV-2 spike protein (PDB ID: 7CN9) (11) displayed in Figure 5B was drawn by ChimeraX (12).

Animal study.

All experiments involving live SARS-CoV-2 were performed in an animal BSL-3 facility at the Genomics Research Center, Academia Sinica. The study protocol was approved by the Institutional Animal Care and Use Committee. Female Golden Syrian hamsters, aged 5-7 weeks old were obtained from National Laboratory Animal Center. 100 μL of PBS containing $1x10^5$ $TCID_{50}$ of SARS-CoV-2 was intranasal instilled under intraperitoneal anesthesia with Zoletil 50 (5 mg/kg) at day 0, and the mock-infected hamsters were challenged with 100 μL of PBS. Body weight and clinical signs of the hamsters were monitored daily during the study as a measure of disease progression. Treatment groups were given through oral administration twice a day with the following compounds: mefloquine, nelfinavir, salinomycin, and thioguanine at a dose of 30 mg/kg/day, leaves of Perilla frutescens (200 mg/kg/day), leaves of Mentha haplocalyx (200 mg/kg/day), and extract of Ganoderma luciudum (30 mg/kg/day), while the control group was given an equal volume of drinking water. At day-3 postinfection, all the hamsters were euthanized and the lung tissues were harvested for live viral load measurement by TCID50 assay in Vero E6 cells.

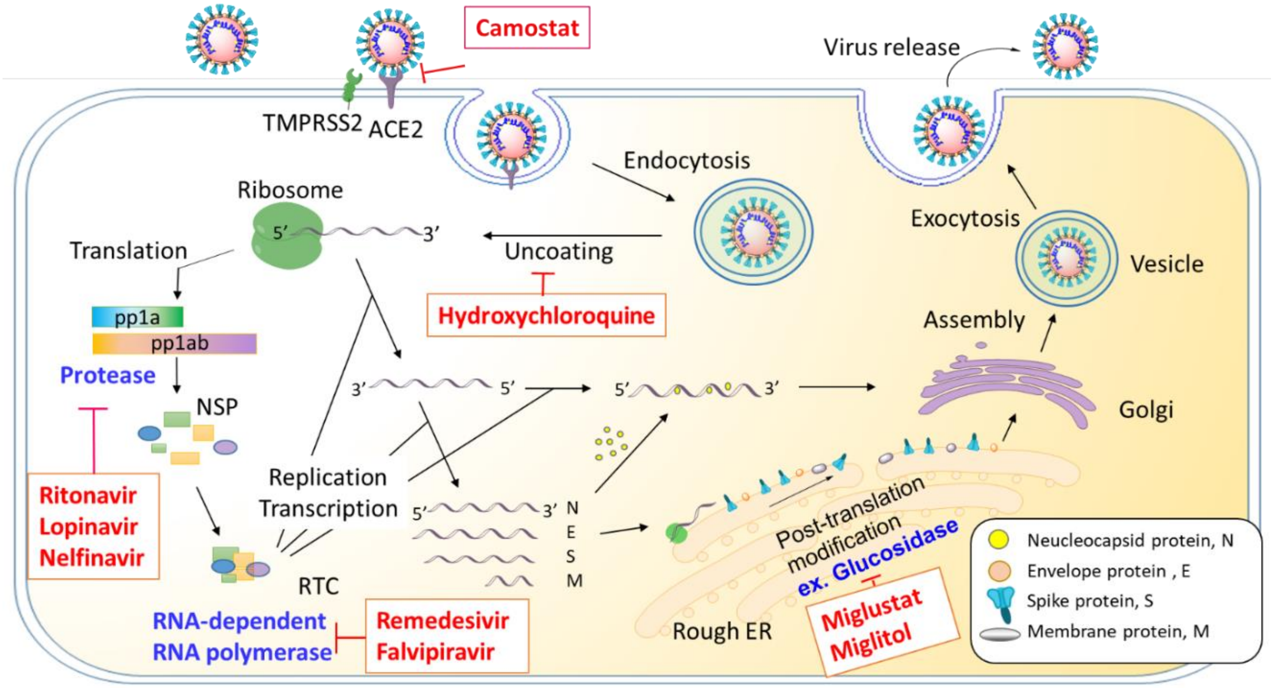

Fig. S1. SARS-CoV-2 life cycle and potential drug targets.

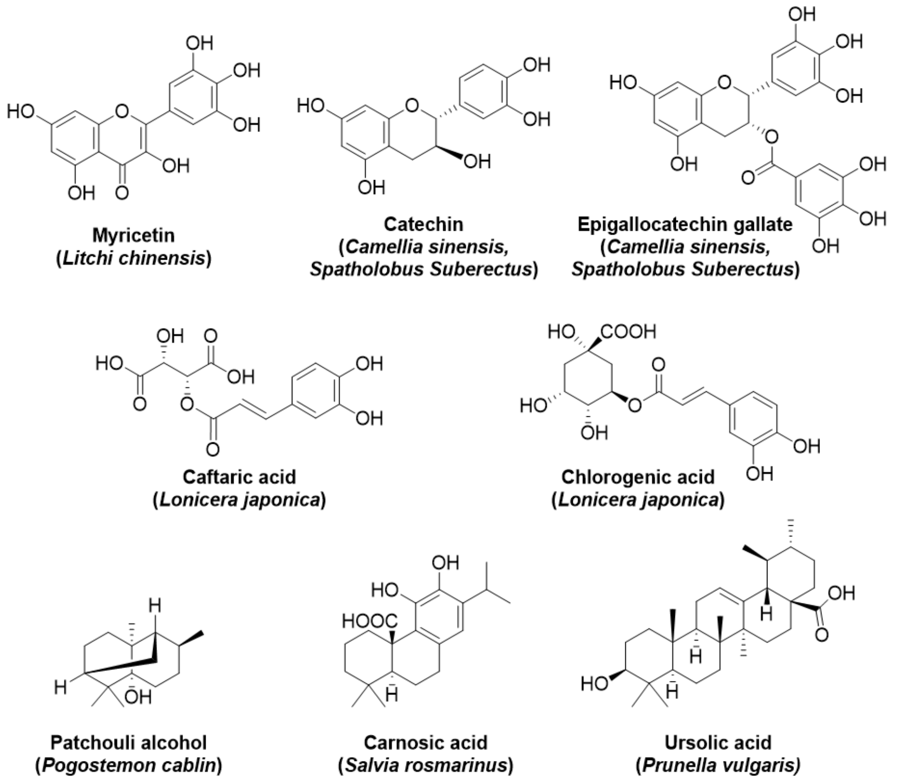

Fig. S2. Representative components from the Chinese herbal medicines and supplements that potentially have anti-infective effects against SARS-CoV-2 in Vero E6 cells. The plant origin related to these components were indicated.

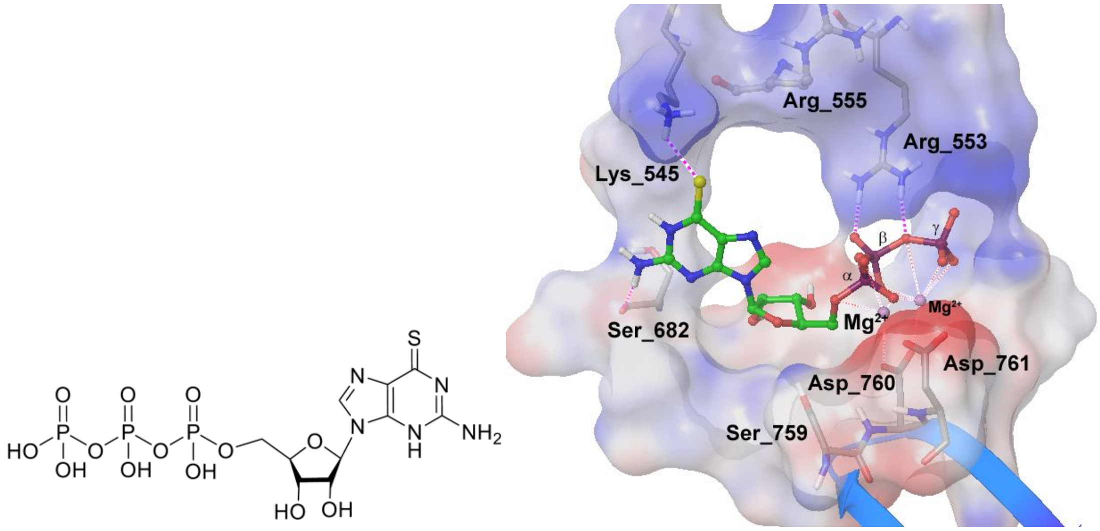

Fig. S3. Structure of TGTP and computer modeling of TGTP binding to SARS-CoV-2 RdRP (PDB coded 7BV2).

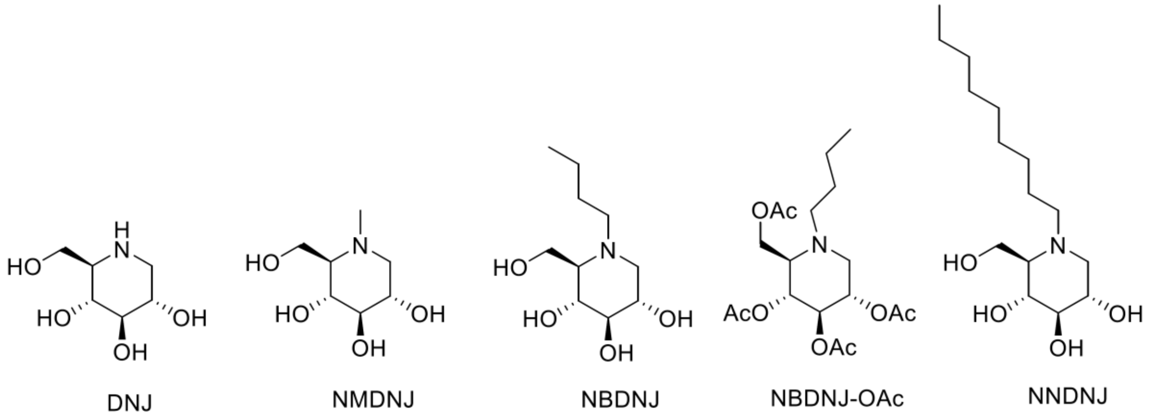

Fig. S4. Structures of α-glucosidase inhibitors for anti-SARS-CoV-2 assay (tested conc. 100 μM).

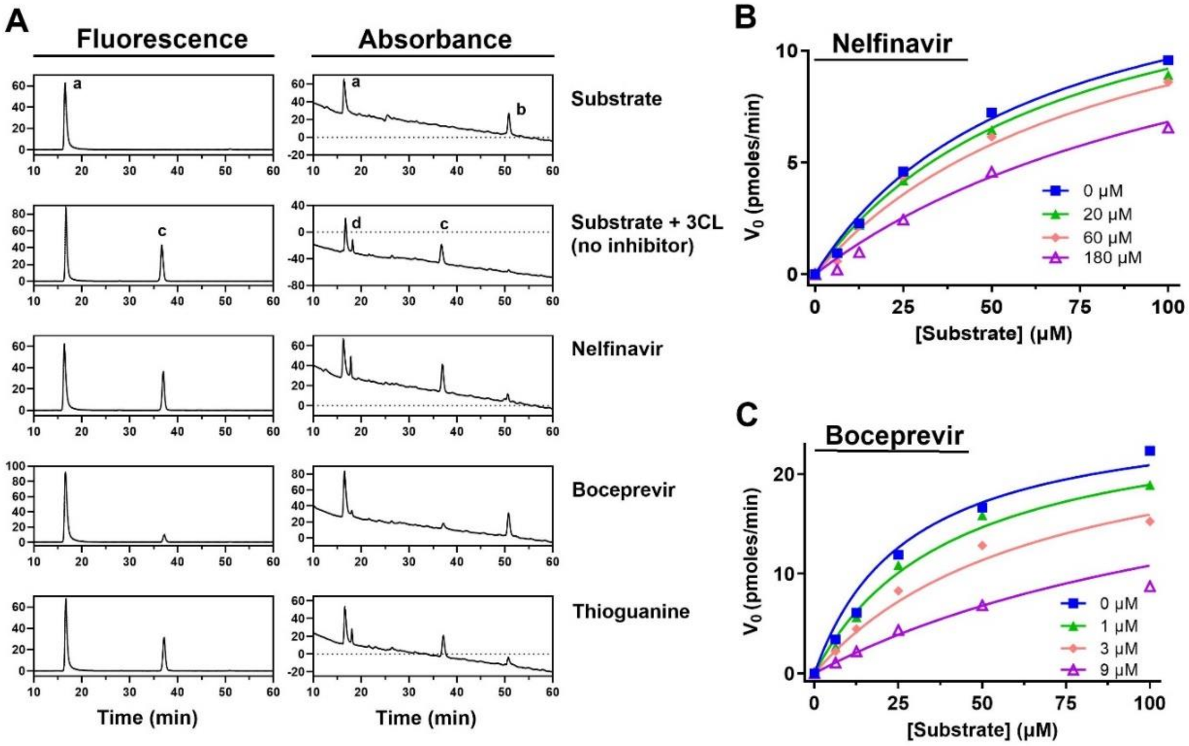

Fig. S5. (A) The HPLC analysis of cleaved substrates by 3CL main protease. Nsp5 was incubated with Fluorescein-AVLQSGFRK(QXL520)-NH_2 in the absence or presence of indicated inhibitors in Tris-HCl (pH 7.0, 20 mM) at 30°C for 30 minutes. After being spiked with 10 μM FAM, the reaction mixture was subjected to high-performance liquid chromatography on a Zorbax C18 column (with a gradient of 5% to 25% acetonitrile containing 0.1% trifluoroacetic acid). The fluorescence was monitored at excitation 480 nm/emission 530 nm and the peptide was monitored at 214 nm. Peak a: Fluorescein; peak b: Fluorescein-AVLQSGFRK(QXL520)-NH_2; peak c: Fluorescein-AVLQ; peak d: GFRK(QXL 520). The K_i determination of nelfinavir (B) and boceprevir (C). Nsp5 was

incubated with Fluorescein-AVLQSGFRK(QXL520)-NH_2 in the absence or presence of indicated inhibitors in Tris-HCl (pH 7.0, 20 mM) at 30°C for 30 minutes.The reaction was monitored with fluorescence (ex480/em530) with a plate reader and the initial velocity was calculated. The curves were fitted using Prism 7.0 (Graphpad). Shown are the representative figures from three independent experiments.

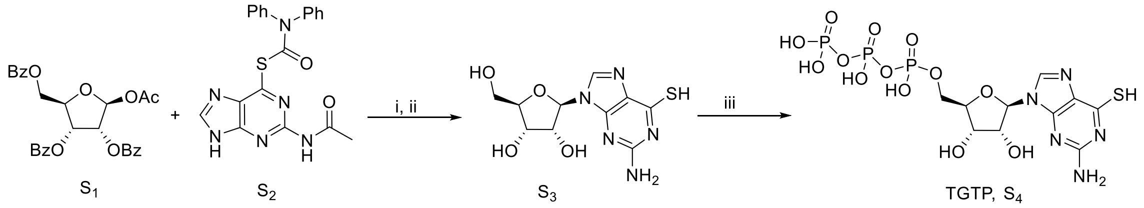

Fig. S6. Preparation of TGTP. (i) BSA, DCE, TMSOTf, Toluene, 80 ^{o}C, 81%; (ii) NH_3, MeOH, 60%; (iii) Proton sponge, $POCl_3$, $(HNBu_3)_2H_2P_2O_7$, $OP(OMe)_3$, NBu_3/DM, 0 ^{o}C**.**

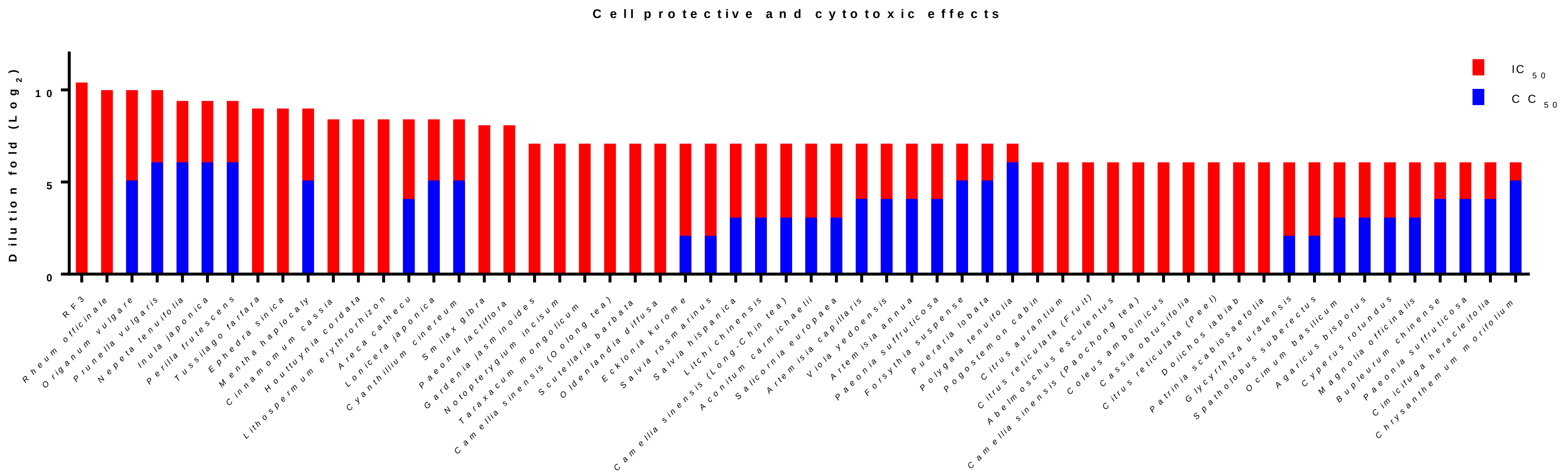

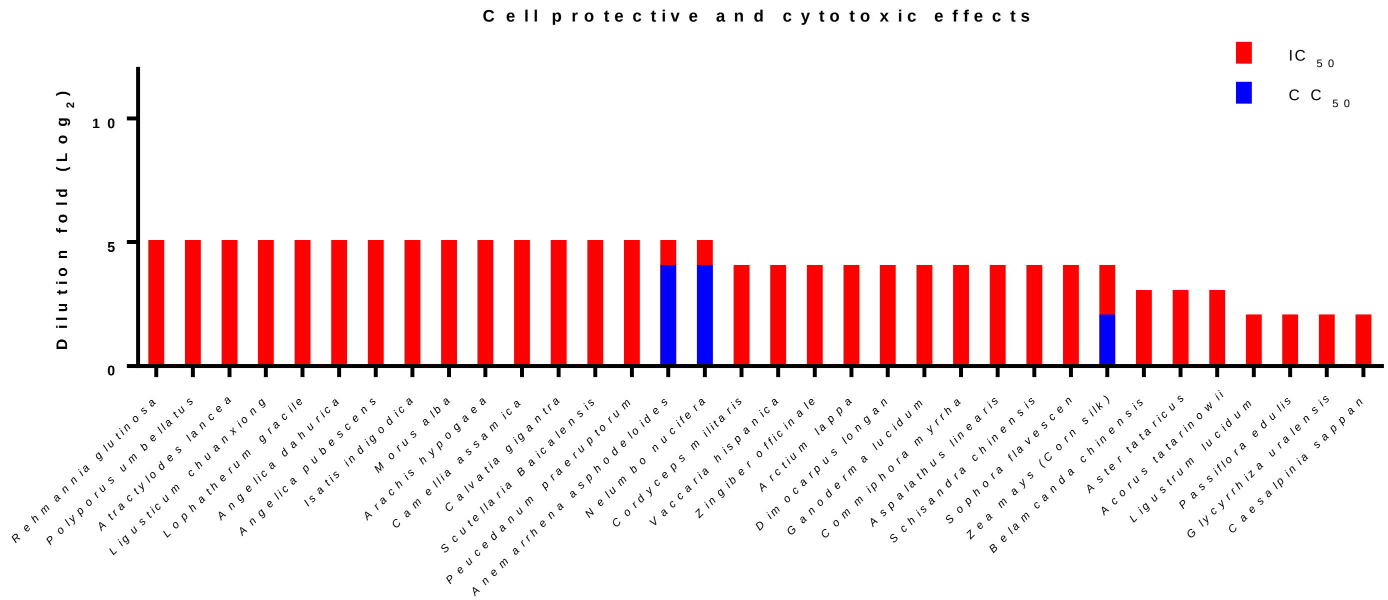

Fig. S7. The anti-SARS-CoV-2 infection effect of Chinese herbal medicines in serial dilutions were represented as Log_2(dilution fold).

Table S1. *In vitro* enzyme inhibition assay against papain-like protease and 3CL main protease of 15 compounds with promising anti-SARS-CoV-2 efficacy.

**Name**	**Inhibition % at 100 uM of compounds[a]**	
	**Papain-like protease (nsp3)**	**3CL main protease (nsp5)**
Nelfinavir **1**	3.5 ± 0.2	21.0 ± 5.1 (10.3[c])
Boceprevir **2**	N.I.[b]	93.8 ± 0.5 (83.6[c])
Thioguanine **3**	N.I.[b]	22.2 ± 5.3 (16.6[c])
Cepharanthine **4**	31.9 ± 1.4	N.I.[b]
Emetine **5**	19.2 ± 0.4	N.I.[b]
Ivermectin **6**	N.I.[b]	N.I.[b]
Moxidectin **7**	N.I.[b]	2.8 ± 4.2
Mefloquine **8**	N.I.[b]	N.I.[b]
Ivacaftor **9**	N.I.[b]	10.9 ± 17.9
Azelnidipine **10**	N.I.[b]	2.0 ± 2.7
Penfluridol **11**	N.I.[b]	25.6 ± 9.3
Dronedarone **12**	78.6 ± 0.3	41.5 ± 1.8 (28.7[c])
Salinomycin **13**	9.4 ± 3.0	N.I.[b]
Monensin **14**	N.I.[b]	N.I.[b]
Maduramicin **15**	N.I.[b]	N.I.[b]

[a]The inhibition percentage was calculated from 3 independent experiments using FRET-based enzymatic assays; [b]No inhibition; [c]The inhibition percentage in parentheses was calculated from the production of substrate based on HPLC analysis.

Table S2. Traditional Chinese herbal medicines and *in vitro* anti-SARS-CoV-2 assay. 1 gram of grounded herbal medicine was extracted by water (20 mL) at room temperature and then centrifuged. The supernatant (100 μL) was combined with 100 μL DMEM medium. Vero E6 cells were treated with a series of dilution (2-fold dilution) of the extract mixture for 2 h and then SARS-CoV-2 (10 $TCID_{50}$ per well) was added. The cytopathic morphology of the cells was examined by using an inverted microscope at 72 h. The anti-infective effect of herbal extracts was highlighted in green (+) and those with cytotoxicity were highlighted in grey (c). [a]Indicating the highest dilution fold that showed the antiinfective or cytotoxic effect.

English Name	Latin Name	Dilution folds					
		4X	8X	16X	32X	64X	128X (max dilution)
Coptis Rhizome	*Coptis chinensis* Franch.	x	x	x	x	x	x
Turmeric Rhizome	*Curcuma longa* L.	x	x	x	x	x	x
Ginseng	*Panax ginseng* C.A.Mey.	x	x	x	x	x	x
Pueraria Root	*Pueraria lobata* (Willd.) Ohwi	c	c	c	c	+	+
Indian Bread	*Poria cocos* (Schwein.) F.A. Wolf	x	x	x	x	x	x
Patchouli	*Coleus amboinicus* Lour.	+	+	+	+	+	x
Mongolian Dandelion Herb	*Taraxacum mongolicum* Hand.-Mazz.	+	+	+	+	+	+
Puerh tea	*Camellia assamica* (Mast.) Chang	+	+	+	+	x	x
Hawthorn fruit	*Crataegus pinnatifida* Bunge	x	x	x	x	x	x
Liquorice root and Rhizome	*Glycyrrhiza uralensis* Fisch.	c	+	+	+	+	x
Fleeceflower root	*Polygonum multiflorum* Thunb.	+	x	x	x	x	x
Peony root	*Paeonia lactiflora* Pall.	x	x	x	x	x	x
Chinese Angelica root	*Angelica sinensis*	x	x	x	x	x	x
Agaricus blazei murill	*Agaricus subrufescens* Peck	x	x	x	x	x	x

Polygala root	*Polygala tenuifolia* Willd.	c	c	c	c	c	+
Paochong tea	*Camellia sinensis* var. *sinensis*	+	+	+	+	+	x
Maitake	*Grifola frondosa* (Dicks.) Gray	x	x	x	x	x	x
Mushroom	*Lentinus edodes*	x	x	x	x	x	x
White beech mushroom	*Hypsizygus tessellatus* (Bull.) Singer	x	x	x	x	x	x
Cremini mushroom	*Agaricus bisporus* (J.E.Lange) Imbach	c	c	+	+	+	x
Dendrobium huoshanense	*Dendrobium huoshanense* C.Z.Tang et S.J.Cheng	x	x	x	x	x	x
Black fungus	*Auricularia polytricha* (Mont.) Sacc.	x	x	x	x	x	x
Samphire	*Salicornia europaea* L.	c	c	+/-	+	+	+
Long-Chin tea	*Camellia sinensis* var. *sinensis*	c	c	+	+	+	+
Matsutake	*Tricholoma matsutake* (Ito. et Imai) Sing.	x	x	x	x	x	x
King oyster mushroom	*Pleurotus eryngii* (DC.) Quél.	x	x	x	x	x	x
Shiro-shimeji	*Hypsizygus tessellatus* (Bull.) Singer	x	x	x	x	x	x
Brown beech mushroom	*Hypsizygus tessellatus* (Peck) H.E. Bigelow	x	x	x	x	x	x
White fungus	*Tremella fuciformis* Berk.	x	x	x	x	x	x
Ecklonia	*Ecklonia kurome* Okam.	c	+	+	+	+	+
Rooibos tea	*Aspalathus linearis* (Burm.f.) R. Dahlgren	+	+	+	x	x	x
Oolong tea	*Camellia sinensis* var. *sinensis*	+	+	+	+	+	+
Boat Sterculia seed	*Sterculia lychnophora* Hance	x	x	x	x	x	x
Grosvenor Momordica Fruit	*Siraitia grosvenorii*	x	x	x	x	x	x
Chuanxiong Rhizome	*Ligusticum chuanxiong* Hort.	+	+	+	+	x	x

Garlic	*Allium sativum* L.	x	x	x	x	x	x
Ginger	*Zingiber officinale* Roscoe.	+	+	+	x	x	x
Black pepper	*Piper nigrum* L.	x	x	x	x	x	x
Chilli	*Capsicum frutescens* L.	x	x	x	x	x	x
Basil leaves	*Ocimum basilicum* L.	c	c	+	+	+	x
Astragalus root	*Astragalus membranaceus* (Fisch.) Bunge.	x	x	x	x	x	x
Caterpillar fungus	*Cordyceps militaris* (L.) Fr.	+	+	+	x	x	x
Chrysanthemum	*Chrysanthemum morifolium* (Ramat.) Hemsl.	x	x	x	x	x	x
Red pepper	*Capsicum chinense* Jacq.	x	x	x	x	x	x
White pepper	*Piper nigrum*	x	x	x	x	x	x
Bitter gourd	*Momordica charantia* L.	x	x	x	x	x	x
Rosemary	*Salvia rosmarinus* Spenn.	c	+	+	+	+	+
Gynostemma	*Gynostemma pentaphyllum* (Thunb.) Makino	x	x	x	x	x	x
Cassia Twig	*Cinnamomum cassia* (L.) J.Presl	+	+	+	+	+	+(320X)[a]
Peony root	*Paeonia lactiflora* Pall.	c	c	c	+	+	x
Apricot delight	*Prunus armeniaca* L.	x	x	x	x	x	x
Prepared Monkshood Daughter root	*Aconitum carmichaelii* Debx.	c	c	+	+	+	+
Dan-shen root	*Salvia miltiorrhiza* Bunge	x	x	x	x	x	x
Perilla fruit	*Perilla frutescens* (L.) Britton	x	x	x	x	x	x
Safflower	*Carthamus tinctorius* L.	x	x	x	x	x	x
Tendrilleaf Fritillary Bulb	*Fritillaria cirrhosa* D. Don.	x	x	x	x	x	x
Peach Kernel	*Prunus persica* (L.) Batsch.	x	x	x	x	x	x
Costus root	*Aucklandia lappa* Decne.	x	x	x	x	x	x
Motherwort fruit	*Leonurus heterophyllus* Sweet	x	x	x	x	x	x

Curcuma root	*Curcuma longa* L.	x	x	x	x	x	x
Malaytea Scurfpea fruit	*Psoralea corylifolia* L.	x	x	x	x	x	x
Schisandra fruit	*Schisandra chinensis* (Turcz.) Baill.	+	+	+	x	x	x
Cucurbitaceae fruit	*Trichosanthes kirilowii* Maxim.	x	x	x	x	x	x
Chia seed	*Salvia hispanica* L.	c	c	+	+	+	+
Dragon's blood	*Daemonorops draco* (Willd.) Blume	x	c	c	x	x	x
Platycodon root	*Platycodon grandiflorus* (Jacq.) A. DC.	x	x	x	x	x	x
Tabasheer	*Bambusa textilis* McClure	x	x	x	x	x	x
Bamboo Shavings	*Bambusa tuldoides* Munro	x	x	x	x	x	x
Myrrh	*Commiphora myrrha* Engl.	+	+	+	x	x	x
Hiraute Shiny Bugleweed herb	*Eupatorium formosanum* Hayata	x	x	x	x	x	x
Sappan wood	*Caesalpinia sappan* L.	+	x	x	x	x	x
Wolfberry fruit	*Lycium chinense* Mill.	x	x	x	x	x	x
Jackinthepulpit Tuber	*Arisaema erubescens* (Wall.) Schott	x	x	x	x	x	x
Spatholobus Root	*Spatholobus suberectus* Dunn	c	+	+	+	+	x
Cowherb seed	*Vaccaria hispanica* (Mill.) Rauschert	+	+	+	x	x	x
Eggplant	*Solanum melongena* L.	x	x	x	x	x	x
Okra	*Abelmoschus esculentus* (L.) Moench	x	x	x	x	x	x
Okra peel	*Abelmoschus esculentus* (L.) Moench	x	x	x	x	x	x
Longan fruit	*Dimocarpus longan* Lour.	x	x	x	x	x	x
Passion fruit peel	*Passiflora edulis* Sims	+	x	x	x	x	x
White Pigeon Peas.	*Cajanus cajan* (L.) Millsp.	Δ	+	Δ	x	x	x
Pigeon pea peel	*Cajanus cajan* (L.) Millsp.	x	x	x	x	x	x
Aloe	*Aloe barbadensis* Miller	x	x	x	x	x	x

Dry ginger	*Zingiber officinale* Roscoe.	x	x	x	x	x	x
Okra seed	*Abelmoschus esculentus* (L.) Moench	+	+	+	+	+	x
Avocado seed	*Persea americana* Mill.	c	+	Δ	x	x	x
Passion fruit	*Passiflora edulis* Sims	x	x	x	x	x	x
Kiwifruit seed	*Actinidia deliciosa*	x	x	x	x	x	x
Pigeon Peas.	*Cajanus cajan* (L.) Millsp.	x	x	x	x	x	x
Awkeotsang	*Ficus pumila* var. awkeotsang (Makino) Corner	x	x	x	x	x	x
Bitter melon	*Momordica charantia*	x	x	x	x	x	x
Bitter melon membrane	*Momordica charantia*	x	x	x	x	x	x
Bitter melon seed	*Momordica charantia*	x	x	x	x	x	x
Kakorot seed	*Momordica charantia* Linn. var. *abbreviata* Seinge	x	x	x	x	x	x
Pomelo seed	*Citrus maxima*	c	c	x	x	x	x
Litchi chinensis seed	*Litchi chinensis*	c	Δ	+	+	+	+
Pomegranate peel	*Punica granatum*	x	x	x	x	x	x
Roselle	*Hibiscus sabdariffa*	x	x	x	x	x	x
Green pepper seed	*Capsicum annuum* var. *grossum*	x	x	x	x	x	x
Pomelo peel	*Citrus maxima*	x	x	x	x	x	x
Litchi chinensis Shell	*Litchi chinensis*	x	x	x	x	x	x
Pomegranate	*Punica granatum*	x	x	x	x	x	x
Roselle seed	*Hibiscus sabdariffa*	x	x	x	x	x	x
Apple peel	*Malus pumila* Mill.	x	x	x	x	x	x
Apple seed	*Malus pumila* Mill.	x	x	x	x	x	x
Tangerine peel	*Citrus reticulata*	x	x	x	x	x	x
Sunflower Seed	*Helianthus annuus*	x	x	x	x	x	x
Corn shell	*Zea mays* L.	x	x	x	x	x	x

Baby corn	*Zea mays* L.	x	x	x	x	x	x
Corn silk	*Zea mays* L.	Δ	+	+	x	x	x
Peanut shell	*Arachis hypogaea* L.	x	x	x	x	x	x
Cabbage	*Brassica oleracea* var. *capitata*	x	x	x	x	x	x
Longan Shell	*Dimocarpus longan* Lour.	+	+	+	x	x	x
Sunflower Seed Shell	*Helianthus annuus*	x	x	x	x	x	x
Peanut	*Arachis hypogaea* L.	x	x	x	x	x	x
Peanut membrane	*Arachis hypogaea* L.	+	+	+	+	x	x
Lotus Plumule	*Nelumbo nucifera* Gaertn.	c	c	c	+	x	x
Morinda Root	Morinda officinalis F.C.	x	x	x	x	x	x
Chinese Yam	*Dioscorea opposita* Thunb.	x	x	x	x	x	x
Trogopterus Dung	*Trogopterus xanthipes* Milne-Edwards	x	x	x	x	x	x
Agastache Herb	*Pogostemon cablin* (Blanco) Benth.	+	+	+	+	+	x
Heartleaf Houttuynia Herb	*Houttuynia cordata* Thunb	+	+	+	+	+	+(320X)[a]
Pinellia Tuber	*Pinellia ternata* (Thunb.) Breitenb.	x	x	x	x	x	x
Mulberry Root Bark	*Morus alba* L.	+	+	+	+	x	x
Glossy Privet Fruit	*Ligustrum lucidum* W.T. Aiton	+	x	x	x	x	x
Curcuma zedoaria	*Curcuma phaeocaulis* Val.	x	x	x	x	x	x
Tangerine Peel	*Citrus reticulata* Blanco	+	x	x	x	x	x
Common Burreed Rhizome	*Sparganium stoloniferum* Buch.-Ham.	x	x	x	x	x	x
Frankincense	*Boswellia carterii* Birdw.	x	x	x	x	x	x
Twotooth Achyranthes Root	*Achyranthes bidentata* Bl.	x	x	x	x	x	x
Perilla	*Perilla frutescens* (L.) Britton	c	c	c	c	c	+(640X)[a]
Honeysuckle flower Bud	*Lonicera japonica*	c	c	c	c	+	+(320X)[a]
Fineleaf Nepeta herb	*Nepeta tenuifolia* (Benth.)Briq.	c	c	c	c	c	+(640X)[a]

Chrysanthemum flower	*Chrysanthemum morifolium* Ramat.	c	c	c	c	+	x
Dahurian Angelica Root	*Angelica dahurica F.*	+	+	+	+	x	x
Ephedra Herb	*Ephedra sinica* Stapf	+	+	+	+	+	+(480X)[a]
Agaric	*Polyporus umbellatus* (Pers) Fries	+	+	+	+	x	x
Blackberry-lily Rhizome	*Belamcanda chinensis* DC.	+	+	x	x	x	x
Forsythia Fruit	*Forsythia suspense* Vahl	c	c	c	c	c	+
Atractylodes Rhizome	*Atractylodes lancea* DC.	+	+	+	+	x	x
Skullcap Herb	*Scutellaria barbata* D. Don	+	+	+	+	+	+
Radix Glycyrrhizae Preparata	*Glycyrrhiza uralensis* F.	+	x	x	x	x	x
Anemarrhena Rhizome	*Anemarrhena asphodeloides* Bunge.	c	c	c	+	x	x
Scutellaria Root	*Scutellaria Baicalensis* Georgi	+	+	+	+	x	x
Immature Bitter Orange	*Citrus aurantium L.*	+	+	+	+	+	x
Senna obtusifolia	*Cassia obtusifolia* L.	+	+	+	+	+	x
Bupleurum Root	*Bupleurum chinense* DC.	c	c	c	+	+	x
Rhubarb	*Rheum officinale*	+	+	+	+	+	+(960X)[a]
Tatarian Aster Root and Rhizome	*Aster tataricus* L.f.	+	+	x	x	x	x
Prunella Spike	*Prunella vulgaris* L.	c	c	c	c	c	+(960X)[a]
Pepperweed Seed Tansymustard Seed	*Lepidium opetalum* Willd.	x	x	x	x	x	x
Coltsfoot Flower Bud	*Tussilago farfara* L.	+	+	+	+	+	+(480X)[a]
Tangerine Peel	*Citrus reticulata*	+	+	+	+	+	x
Amur Corktree Bark	*Phelloendron amurense* Rupr.	x	x	x	x	x	x
Oriental Waterplantain Tuber	*Alisma orientalis* Juzep.	x	x	x	x	x	x

Largehead Atractylodes Rhizome	*Atractylodes macrocephala* Koidz.	x	x	x	x	x	x
Manchurian Wildginger Herb	*Asarum heterotropides*	x	x	x	x	x	x
Divaricate Saposhnikovia	*Saposhnikovia divaricata* Schischk.	x	x	x	x	x	x
Chinese Gentian Root	*Gentiana scabra* Bge.	x	x	x	x	x	x
Tatarinow Sweerflag Rhizome	*Acorus tatarinowii* Schott.	+	+	x	x	x	x
Sophora	*Sophora flavescen* Ait.	+	+	+	x	x	x
Lucid Ganoderma	*Ganoderma lucidum* (Leyss.ex Fr.) Karst.	+	+	+	x	x	x
Great Burdock Fruit	*Arctium lappa* L.	+	+	+	x	x	x
Whiteflower Hogfennel Root	*Peucedanum praeruptorum* Dunn.	+	+	+	+	x	x
Puffball	*Calvatia gigantra* (Batsh ex pers.) Loly	+	+	+	+	x	x
Adhesive Rehmannia Root	*Rehmannia glutinosa* Libosch.	+	+	+	+	x	x
Doubleteeth Angelicae Root	*Angelica pubescens* Maxim. F.biserrata Shanet Yuan	+	+	+	+	x	x
Common Lophatherum Herb	*Lophatherum gracile* Brongn.	+	+	+	+	x	x
Indigowoad Leaf	*Isatis indigodica* Fort.	+	+	+	+	x	x
Immature Tangerine Fruit	*Citrus reticulata* Blanco.	+	+	+	+	+	x
White Hyacinth Bean	*Dolichos lablab* L.	+	+	+	+	+	x
Whiteflower Patriniae Herba	*Patrinia scabiosaefolia* Fisch ex Link.	+	+	+	+	+	x
Nutgrass Galingale Rhizome	*Cyperus rotundus* L.	c	c	+	+	+	x
Officinal Magnolia Bark	*Magnolia officinalis* Rehd. et Wils.	c	c	+	+	+	x
Skunk Bugbane Rhizome	*Cimicifuga heracleifolia* Kom.	c	c	c	+	+	x
Common Gardenia Fruit	*Gardenia jasminoides* Ellis.	+	+	+	+	+	+
Incised Notopterygium Rhizome and Root	*Notopterygium incisum* Ting ex H. T. Chang	+	+	+	+	+	+

Spreading Hedyotis Herb	*Oldenlandia diffusa* (Willd.) Roxb.	+	+	+	+	+	+
Sweet Wormwood Herb	*Artemisia annua* L.	c	c	c	+	+	+
Capillary Wormwood Herb	*Artemisia capillaris* Thunb.	c	c	c	+	+	+
Tree Peony Rootbark	*Paeonia suffruticosa* Andr.	c	c	c	+	+	+
Manchurian Violet	*Viola yedoensis* Makino.	c	c	c	+	+	+
Red Paeoniae Trichocarpae	*Paeonia lactiflora* Pall.	+	+	+	+	+	+(256X)[a]
Glabrous Greenbrier Rhizome	*Smilax glbra* Roxb.	+	+	+	+	+	+(256X)[a]
Redroot Gromwell	*Lithospermum erythrorhizon* Sieb. et Zucc.	+	+	+	+	+	+(320X)[a]
Betelnutpalm Seed	*Areca cathecu* L.	c	c	c	+	+	+(320X)[a]
Little ironweed	*Cyanthillium cinereum*	c	c	c	c	+	+(320X)[a]
Wild Mint Herb	*Mentha haplocalyx*	c	c	c	c	+	+(480X)[a]
Japanese Inula Flower	*Inula japonica* Thunb.	c	c	c	c	c	+(640X)[a]
Origani Vulgaris Herba	*Origanum vulgare* L.	c	c	c	c	+	+(960X)[a]

SI References

1. S. F. Liao *et al.*, Immunization of fucose-containing polysaccharides from Reishi mushroom induces antibodies to tumor-associated Globo H-series epitopes. *Proc. Natl. Acad. Sci. U. S. A.* 110, 13809-13814 (2013).
2. J. J. Shie *et al.*, Inhibition of the severe acute respiratory syndrome 3CL protease by peptidomimetic alpha,beta-unsaturated esters. *Bioorg Med Chem* 13, 5240-5252 (2005).
3. H. S. Hillen *et al.*, Structure of replicating SARS-CoV-2 polymerase. *Nature* 584, 154-156 (2020).
4. R. Huey, G. M. Morris, A. J. Olson, D. S. Goodsell, A semiempirical free energy force field with charge-based desolvation. *J. Comput. Chem.* 28, 1145-1152 (2007).
5. L. Zhang *et al.*, Crystal structure of SARS-CoV-2 main protease provides a basis for design of improved α-ketoamide inhibitors. *Science* 368, 409-412 (2020).
6. W. Yin *et al.*, Structural basis for inhibition of the RNA-dependent RNA polymerase from SARS-CoV-2 by remdesivir. *Science* 368, 1499-1504 (2020).
7. T. A. Steitz, J. A. Steitz, A general two-metal-ion mechanism for catalytic RNA. *Proc. Natl. Acad. Sci. U. S. A.* 90, 6498-6502 (1993).
8. D. Shivakumar, E. Harder, W. Damm, R. A. Friesner, W. Sherman, Improving the Prediction of Absolute Solvation Free Energies Using the Next Generation OPLS Force Field. *J. Chem. Theory Comput.* 8, 2553-2558 (2012).
9. J. R. Greenwood, D. Calkins, A. P. Sullivan, J. C. Shelley, Towards the comprehensive, rapid, and accurate prediction of the favorable tautomeric states of drug-like molecules in aqueous solution. *J. Comput. Aided Mol. Des.* 24, 591-604 (2010).
10. K. Katoh, J. Rozewicki, K. D. Yamada, MAFFT online service: multiple sequence alignment, interactive sequence choice and visualization. *Brief. Bioinform.* 20, 1160-1166 (2019).
11. Y. M. Liu *et al.*, A Carbohydrate-Binding Protein from the Edible Lablab Beans Effectively Blocks the Infections of Influenza Viruses and SARS-CoV-2. *Cell Rep.* 32, 108016 (2020).
12. T. D. Goddard *et al.*, UCSF ChimeraX: Meeting modern challenges in visualization and analysis. *Protein Sci.* 27, 14-25 (2018).
